# Ergonomic Considerations in the Incidence of CTS in College of Dentistry, King Khalid University, Abha – Kingdom of Saudi Arabia

**DOI:** 10.3290/j.ohpd.a44031

**Published:** 2020-07-04

**Authors:** Sultan Mohammed Kaleem, Shaik Mohammed Asif, Mohammad Zahir Kota, Tanveer Alam, Hassan Assiri, Meer Zakirullah

**Affiliations:** b Assistant Professor, Department of Diagnostic Sciences and Oral Biology, College of Dentistry/King Khalid University, Abha – Kingdom of Saudi Arabia. Contributed to the design and concept of the study, framed the results.; a Assistant Professor, Department of Diagnostic Sciences and Oral Biology, College of Dentistry/King Khalid University, Abha – Kingdom of Saudi Arabia. Contributed to the design and concept of the study; conducted clinical tests and documented the results; edited the manuscript.; c Assistant Professor, Department of Oral Maxillo Facial Surgery, College of Dentistry/King Khalid University, Abha – Kingdom of Saudi Arabia. Conducted the clinical tests, and documented the results.; d Assistant Professor, Department of Diagnostic Sciences and Oral Biology, College of Dentistry/King Khalid University, Abha – Kingdom of Saudi Arabia. Revised the manuscript, conducted the clinical tests.; e Demonstrator, Department of Diagnostic Sciences and Oral Biology, College of Dentistry/King Khalid University, Abha – Kingdom of Saudi Arabia. Framed the statistical analysis for the study.; f Assistant Professor, Department of Pediatric Dentistry and Orthodontic Sciences College of Dentistry, King Khalid University, Abha – Kingdom of Saudi Arabia. Reviewed and edited the manuscript.

**Keywords:** carpel tunnel syndrome, ergonomics, Phalen’s test, Tinsel’s sign

## Abstract

**Purpose:**

Ergonomics in dentistry poses some challenges to dentists and may require considerable concentration and attention to detail. This research enables early recognition and prevention of common ergonomic-related conditions, such as carpel tunnel syndrome, back pain and neck pain. The purpose of this study was to determine the prevalence of ergonomic-related problems concerning carpel tunnel syndrome (CTS) and to know the efficacy of independent and combined clinical tests used in diagnosing it.

**Materials and Methods:**

Initially the participants were instructed to complete a self-administered questionnaire regarding the severity of symptoms of their hands on a hand–wrist diagram and a visual analogue scale. The principle investigator evaluated all questionnaires independently and four clinical tests were used on both hands in a systematic (non-randomised) order for subjects who had symptoms. Those with residual symptoms that exceeded beyond 1 min interval were identified and controlled for the statistical analyses.

**Results:**

The most common symptom noted in the study group was tingling and numbness of fingers (66.46%) followed by neck pain (66.34%). 29.26% of subjects reported moderate difficulty in typing and driving vehicles, whereas 26.82% subjects felt moderate difficulty in grasping and carrying shopping bags. 61.94% of subjects with symptoms spent more than 1 h daily of their free time on mobile phones or other smart devices. Individually, in our study the Tinsel’s sign stood out as ineffective in ruling out CTS when compared with Phalen’s test. Combination tests like Phalen’s test and compression tests are confirmatory to CTS diagnosis and 66.34 % of the research group were hence diagnosed for CTS.

**Conclusions:**

A positive criteria for CTS, neck and shoulder pain is identified in our study as being due to long-term use of mobile devices. Further, combination tests like Phalen’s with pressure provocation tests proved accurate in conforming CTS. Future research is needed to confirm the diagnostic utility of these independent and combined clinical tests in less prevalent settings, including general dental practitioners and occupational worksites.

**Trial registration:**

The current study is registered in King Khalid University, College of dentistry ethical committee SRC/REG/2016-17/107.

Carpal tunnel syndrome (CTS) is due to compression of the median nerve along the wrist.^22^ In acute and mild cases, carpal tunnel syndrome does not demonstrate any signs and symptoms. Various studies have attempted to establish a gold standard from clinical symptoms, but with little success. Though there is no gold standard for diagnosing carpal tunnel syndrome, various studies have used a patient’s rating of severity of a single symptom as an outcome measure. This is considered by many researchers to be valuable in accurately diagnosing carpal tunnel syndrome.^20^ Katz et al^17,18^ suggested that clinical tests are useful in diagnosing carpal tunnel syndrome due to their financial savings, compared with more extensive costs with respect to time and equipment afforded by electrodiagnostic evaluation.^17,18^ Beside diagnostic benefit, currently there are several convenient clinical tests used to evaluate patients suffering from carpal tunnel syndrome. The list of clinical tests used to diagnose carpal tunnel syndrome is extensive and include self-administered symptom reporting diagrams, symptom severity functional status questionnaire, Phalen’s wrist flexion test, wrist extension test, Tinsel’s sign and pressure provocative test.^13^ Furthermore, interactive microcomputer programs for clinical screening of carpal tunnel syndrome have demonstrated a successful degree of accuracy.^8,13^

The literature has reported a wide range of utility measures including sensitivity and specificity for diagnosing carpal tunnel syndrome.^4^ Independent and combined clinical tests continues to be a popular component in decision-making. Visual analogue scales for pain in particular have been a popular outcome measure in many trials of unconventional treatments for CTS, but a statistically significant number of patients with CTS report no pain, either before or after treatment. In order to capture the full range of different symptoms reported by CTS patients, a better approach is to combine this sort of measure for several different clinical tests and add them together to obtain a summary score. The clinical tests conducted can be individual tests or a combination of tests for assessment of CTS like Phalen’s test, reverse Phalen’s test, Durken’s carpel compression test, pressure provocative tests, Tinel’s sign or a combination of any one of the above.^17,18^

Ergonomics is the science of matching working conditions and human capabilities. We must match our tools, equipment, and working methods to our needs in order to perform comfortably and at our best, and learn to recognise conditions that lead to discomfort, implement changes to minimise or eliminate those conditions.^5,11^

Ergonomics in dentistry poses some challenges on dental work and may require considerable concentration and attention to detail.^5,10^ This research enables early recognition and prevention of such ergonomic-related conditions like CTS, back pain and neck pain. The purpose of this study was to determine the prevalence of ergonomic-related problems concerning CTS and to know the efficacy of independent and combined clinical tests used in diagnosing it.

## MATERIALS AND METHODS

Students (Level 6 to Level 12 interns and faculty) working in King Khalid University College of Dentistry were the target population for the study. It was conducted at the College of Dentistry King Khalid University in the academic calendar year 2016–2017 and carried over until mid-2018. The participants consisted of 70.8% males with mean age of 40–50 y, and 29.2% females with mean age of 25–40 y (Table 1).

**Table 1:** Mean age/sex of total group 1242

Mean age	Sex ratio M:F 8:3	Percentage
40–50 + SD 5.5	Males 880	70.8%
25–40 + SD 5.5	Females 362	29.2%

**Fig 1:**
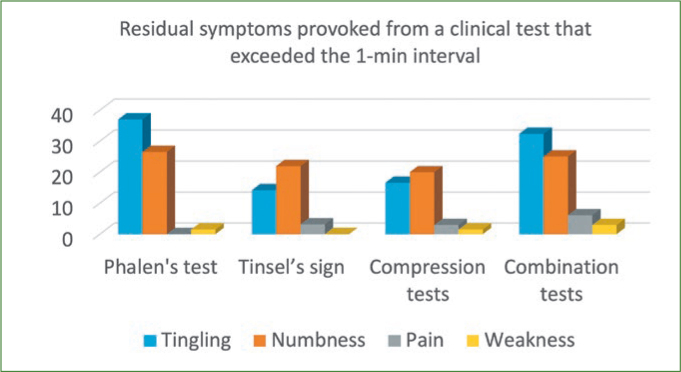
Comparison of four clinical tests for CTS in virtue of symptoms.

**Fig 2:**
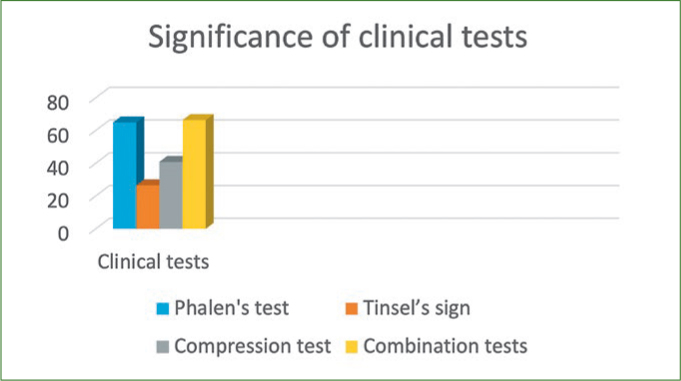
Significance of clinical tests to rule out CTS.

**Fig 3:**
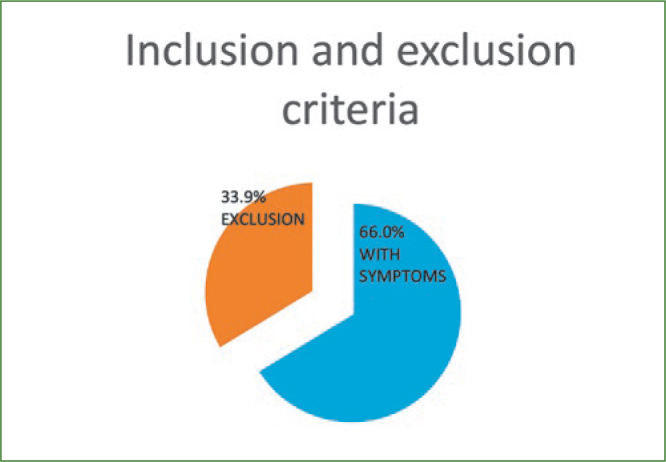
Inclusion and exclusion of research group.

**Fig 4:**
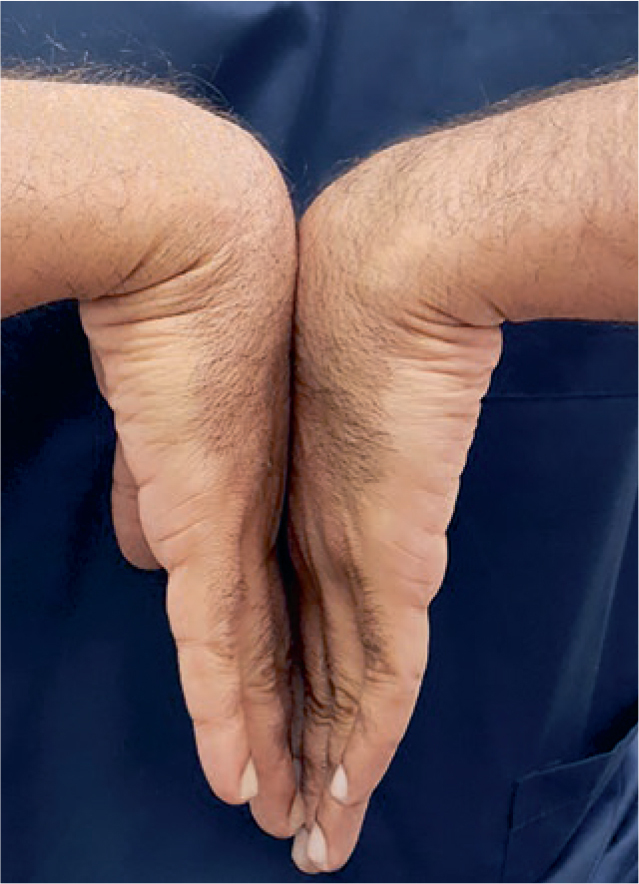
Hand wrist image (Phalen’s test).

**Fig 5:**
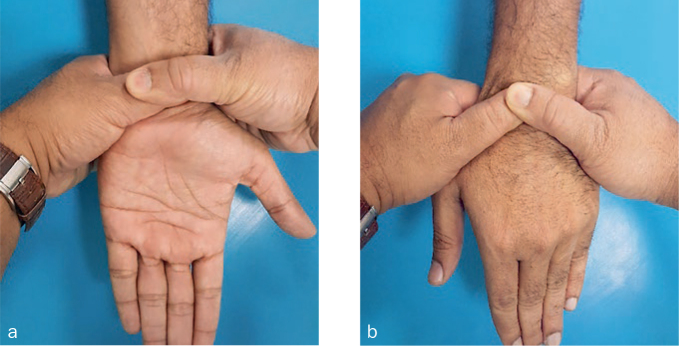
Hand wrist image (pressure provocative test).

**Fig 6:**
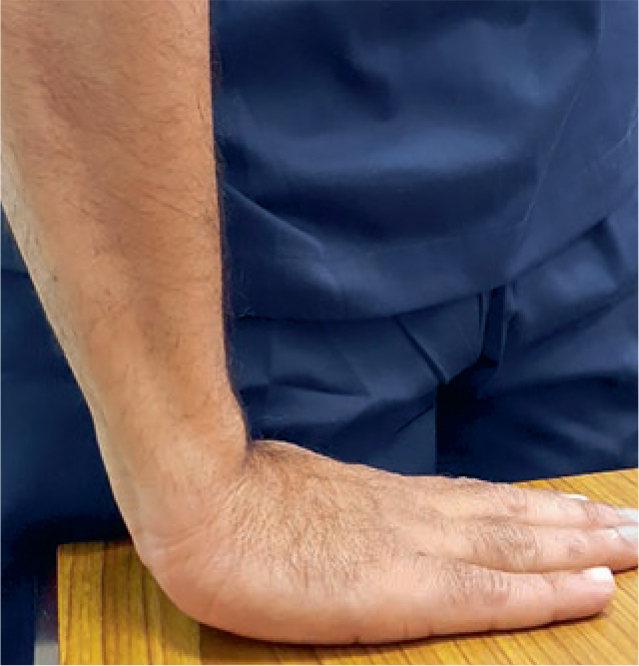
Hand Wrist image (combination test).

A probability sampling method similar to stratified random sampling was implemented and the sample size was determined by the formula (n = z2*P(1-P)/d2). Accordingly, the sample size estimated was 1196, which was rounded to 1242. Participants willing to participate in the study were informed and their consent was taken on a separate consent form and thus included in the study. Subjects not willing to participate, with no symptoms, who were uncooperative, had previous history of joint disorders, and medically compromised subjects were all excluded from the study. Questionnaires pertaining to demographics, symptoms and ergonomics were used as data collection instruments along with diagnostic hammer, gloves, visible LED light and pressure gauge. Ethical clearance for conduction of this study in outpatient clinics was requested from the scientific research committee of the College of Dentistry, King Khalid University (SRC/REG/2016-17/107). The first part of the research was based upon the distribution of a self-administered demographic, symptom reporting questionnaire for severity and functional status by drawing in the appropriate symbols for pain, tingling, decreased sensation and numbness on a hand diagram.^17^ The following rating system, as outlined by Katz et al^18^, was used to assess subjects’ hand symptom questionnaire. Participants who had symptoms like CTS were assessed further clinically with four module tests (Figs 1–3) like Phalen’s test (Fig 4) Tinsel’s sign,^15,26^ compression test (pressure provocative test^13,14^ (Fig 5) and any combination of two tests (Fig 6).

A classic carpal tunnel rating would be indicated if the subject had tingling, numbness or decreased sensation with or without pain in the index, middle or ring finger. An unlikely rating is indicative of a subject with no symptoms.^25^ The principle investigator evaluated the questionnaires independently and classification of each hand diagram was recorded as either classic, probable, possible or unlikely, as reported, in Stirrat’s Symptom Response Diagnostic Report.^17,18^ Four clinical tests were administered on both hands in a systematic (non-randomised) order for subjects who had symptoms as per demographic questionnaire, which include: (i) Phalen’s test; (ii) reverse Phalen’s test; (iii) the pressure provocative test; and (iv) Tinel’s sign by the attending maxillofacial surgeon. A 1-min rest interval was allowed between each clinical test in order to control for residual symptoms that may have lingered as a result of provoking the median nerve. Residual symptoms provoked from the clinical test that exceeded beyond a 1-min interval were identified and controlled for statistical analyses.

### Statistical Analysis

Data obtained and collected was evaluated in Excel 2010 (Microsoft, Redmond, WA, USA) and subjected to required statistics. One-way analysis of variance (ANOVA) was used to determine gender differences for age and McNemar’s chi-square analyses to determine statistically significant differences for sensitivity and specificity of the positive and negative predictive values between the four clinical tests.

## RESULTS

Participants experiencing symptoms in their hands and fingers were asked to complete a symptom-reporting questionnaire by outlining the distribution of their symptoms as suggested by Stirrat^18^. Thus 66% of participants with symptoms were included in the study for further evaluation and the 33.9% without symptoms were excluded (Fig 3).

The most prevalent symptom in the study group was tingling and numbness of fingers in 66.46%, followed by neck pain 66.34% with increased incidence in males compared with females. 8.5% of subjects reported a burning sensation in their fingers (Table 2). Participants with symptoms felt these in a range of mild to moderate intensity at 1.5 on visual analogue scale. 61.70% experienced symptoms in a range of 1 to 2, and 17.07% experienced more severe symptoms in a range of 5 to 6 (Tables 3 and 4). In our study, 92% of symptoms occurred once a day and were present during typing and writing. 5.73% of subjects woke up at night with symptoms, which was not statistically significant in comparison with 94% who did not wake up during the night (Table 5). 80.85% complained of tingling and numbness in any two fingers with mild to moderate intensity in right hand when compared with the left hand (Table 6). The other associated conditions prevailing in study subjects were spondylitis (23.78%) and gout (5.36%); almost 70.80% of subjects were free from any other associated conditions (Table 7). In relation to the difficulty factor faced by the participants, 29.26% participants felt moderate difficulty while typing at a computer and driving a vehicle. 26.82% of participants felt moderate difficulty in both grasping and carrying shopping bags. It was observed in our study that 61.94% of participants with difficulties spent more than 1 h of their free time on mobile phones and other smart devices (Tables 8 and 9). 44.02% of the study group saw 6 to 10 patients per day, and around 52.07% took more than 15 min on each patient. In regard to ergonomic factors in the working environment, it was observed from our study that 5.60% of study subjects felt that the operating light is insufficient for some extended treatment modalities, and 14.51% used direct vision while treating patients, leading to excessive bending or leaning. 68.17% worked in a combination of direct and indirect vision, and keep changing position when they work, which rules out that their symptoms are related to ergonomics, but rather that they spend more than 1 h daily on smart phones and other electronic devices (Table 10). In our study, individual Phalen’s test proved effective in ruling out symptoms of CTS like tingling, numbness and pain in 64.87% subjects, hence proving statistically significant in CTS diagnosis with a p value 0.04 level of statistical significance. Residual symptoms provoked from clinical tests that exceeded a 1 min interval were significantly proven, with a 0.05 level of statistical significance with combination tests, ie, Phalen’s with compression tests, eg, pressure provocative tests (Table 11).

**Table 2:** Prevalent symptom in study group

Symptom	Study subjects		Percentage
	Male	Female	Total
Tingling	215 (26.2%)	57 (6.95%)	272 (33.1%)
Numbness	215 (26.2%)	58 (7.07%)	273 (33.29%)
Neck pain	372 (45.36%)	172(20.97%)	544 (66.34%)
Both tingling & numbness	430 (52.43%)	115 (14.02%)	545 (66.46%)
Burning	25 (3.04%)	45(5.48%)	70 (8.5%)

**Table 3:** VAS (visual analogue scale) inference in relation with intensity and severity of symptoms in study group

VAS scale	1 to 10
Average intensity of symptom felt in range of 1 to 10	1.17
Median of intensity	2.5
Mode	2
SD	1.55

**Table 4:** 

VAS range	Number of subjects	Percentage
1 to 2	506	61.70%
3 to 4	174	21.21%
5 to 6	140	17.07%

**Table 5:** Intensity of pain during activity

Intensity	Number	%	P value
> Once daily	754 (115F/639M)	92%	0.02
Twice daily	44 (M)	5.36%	Not statistically significant
> More than twice in a day	–	0.%	Not statistically significant
> Wakes up at night	47 (M)	5.73%	Not statistically significant
> Did not disturb sleep	770(373F/397M)	94%	0.05
> Felt during driving	56 (M)	6.82%	0.04
> Felt during typing, writing	775 (115F/640M)	92.11 %	0.01
> Symptoms always present	4 (M)	0.48 %	Not statistically significant
> Intermittent symptoms	813(115F/698M)	99.26%	0.05

**Table 6:** Location of symptoms in study group

Symptom	Location/No. of subjects				Severity Scale VAS %
	Left hand	%		Left hand	
Tingling	97	11.82%	Tingling	97	11.82%
Numbness	60	7.31%	Numbness	60	7.31%
Wakes up at night	NIL	0%	Wakes up at night	NIL	0%
Holding or grasping small objects	NIL	0%	Holding or grasping small objects	NIL	0%
TOTAL	157	19.14%	TOTAL	157	19.14%

**Table 7:** Associated conditions in the study group

Associated conditions	No of subjects	Percentage	Location	No of subjects	Percentage
Spondylitis	195(45F/150M)	23.78%	Any two fingers	740 (86F/ 654 M)	90.24%
Gout	44 (M)	5.36%	None	80	9.75%
NIL	581	70.80%			
TOTAL	820	100%		820	100%

**Table 8:** Activities vs difficulty level of study subjects and duration of smart phone usage

Daily activities	Difficulty level	No.	%
Writing/typing	Moderate	64	7.80%
Buttoning clothes	NIL	0	
Holding books	NIL	0	
Grasping	Moderate	135	16.46%
Opening jars	NIL	0	
Carrying bags	Moderate	101	12.31%
Bathing	NIL	0	
Driving vehicles	Moderate	60	7.31%
Both typing and driving	Moderate	240	29.26%
Both grasping and carrying bags	Moderate	220	26.82%
TOTAL		820	99.96%

**Table 9:** Mobile device usage

Total	< 1 h	%	>1 Hr	%
820	312	38.04 %	508	61.94%

**Table 10:** Ergonomic considerations V/s CTS symptoms

	Criteria	Study group	Percentage
No. of patients	1 to 5	184	22.43%
	6 to 10	361	44.02%
Time taken	15min	118	14.39%
	> 15min	427	52.07%
Operator position	Sitting	609	74.26%
	Standing	93	11.34%
	Both	118	14.39%
Status of light	Fair	774	94.39%
	Poor	46	5.60%
Type of vision	Direct	119	14.51%
	Indirect	142	17.31%
	Combination	559	68.17%

**Table 11:** Significance of four clinical tests for CTS in virtue of symptoms

Clinical tests	Symptoms								Total	%	P value
	Tingling		Numbness		Pain		Weakness				
	n = 820	%	n = 820	%	n = 820	%	n = 820	%			
Phalen’s	303	36.95	217	26.46	0	0	12	1.46	532	64.87	0.04
Tinel’s sign	116	14.14	75	21.9	26	3.17	0	0	217	26.46	0.07
Compression	136	16.56	163	19.87	24	2.92	12	1.46	335	40.82	0.06
Combination	265	32.31	205	25	50	6.09	24	2.92	544	66.34	0.05

Combination tests like the Phalen’s test with pressure provocative tests were confirmatory to CTS diagnosis. Hence, 66.34 % of research group in our study were diagnosed.

## DISCUSSION

Dental students and professionals work in one position (static posture) for long periods during a normal day, although they may bend forwards or to the side while working on patients. They may have to stay in one place for a long time rather than moving around frequently, and often use awkward hand and arm postures to gain precision and dexterity to manipulate small dental instruments with force.^9,10,22^

Currently, carpal tunnel syndrome affects over 8 million Americans; almost half of cases result in 31 days or more of work loss.^12^ The relationship between work and CTS occurrence was stressed by previous studies; this causal link is sustained by the difference in its prevalence found among employees in occupations with high physical exposure/awkward posture level versus workers performing low exposure jobs.^3,9,10^ Also, targeted ergonomic interventions succeeded in reducing the number of upper extremity musculoskeletal disorders for workers in hazardous tasks.^1,3^ Although the role of psychosocial factors is not fully assessed, there is strong evidence in literature regarding the relationship between physical exposure and CTS.^7,16^ A statistically significant incidence of carpal tunnel syndrome and other work-related musculoskeletal disorders (WRMSDs) has been recognised in dental practice.^23^ Positive correlations have been found between symptoms of CTS and the number of years worked, number of days worked per week, number and type of procedures. Also, the number of heavy calculus (tartar) deposits identified and treated, patients seen per day, days worked per week, time and years of practice were statistically significant predictors of reported shoulder trouble.^3,19^ Liss^19^ found that the risk of experiencing wrist/hand symptoms increases sharply after 1 year of practice. In our study subjects, symptoms such as tingling (56.46%) and numbness (10.36%) occur once in a day in the right hand and almost always occurred during typing and writing (92.11%) with mild to moderate intensity on visual analogue scale, and around 23.78% suffer from neck and shoulder pain due to spondylitis. The differences in our study in the results of sensitivity and specificity of the assessed provocative tests in patients with CTS are explained by the non-homogenous character of the examined groups of patients, different degrees in progression of the syndrome, as well as various influencing factors. In most publications, the authors did not determine a cause for the syndrome and diagnosed as traumatic or idiopathic CTS in 45%.^24^ The methods for performing clinical tests also seem to play a statistically significant role, especially the percussion test; eg, some use their fingertips, while others perform use a neurological hammer. In our study, a neurological hammer was used to provoke a response. By lightly tapping (percussing) over a nerve to elicit a sensation of tingling or ‘pins and needles’ in the distribution of the nerve, as suggested by Heller, Mossman and Belau^6,15,21^ obtained a positive Tinel sign in 49% using fingertips, while generating up to 79% positive signs with a hammer in the same group of patients.^15,21^ Considering this, Buch-Jaeger and Brüske^6,7^ proposed modified provocative tests with standardised pressure force, which resulted in increased sensitivity in 67% and specificity up to 90%. Gellman et al^14^ in their studies achieved 44% sensitivity and 68% specificity. Based on these studies, one can conclude that the percussion test is characterised by low sensitivity and at the same time, relatively high specificity. In our study, Tinel’s sign, performed in 14.14% of subjects, was not statistically significant to rule out CTS (Table 11). Phalen’s test, which requires flexion of the wrist to cause compression of nerve under the palmar ligament, is a classic provocative test as described in one study by Brüske,^7^ where sensitivity approached 87% and specificity 94%. In our study, residual symptoms provoked from clinical tests that exceeded a 1-min interval were tingling and numbness, which was significantly proven by Phalen’stest and combination tests. Phalen’s test, in combination with pressure compression tests, has proven diagnostically statistically significant in diagnosing CTS. High sensitivity and specificity for Phalen’s test is similar to the results of other authors. Individually, Phalen’s test proved effective in ruling out symptoms of CTS in 64.87% subjects, and hence ranked highly specific in CTS diagnosis. Analysis of selected factors, such as duration of symptoms and gender, did not reveal a statistically significant influence on the results (Table 2).

One interesting finding was made during the study with regard to the influence of the use of mobile phones and other digital devices and the duration of symptoms. 29.26% of subjects in our study group felt moderate difficulty in both typing on a computer and driving vehicles when 26.82% of subjects felt moderate difficulty in both grasping and carrying shopping bags. Almost 61.94% of subjects with difficulty spent more than 1 h daily of their free time on mobile phones or other digital devices (Tables 8 and 9). A study conducted by Aparna Nathan^2^ drew similar conclusions, which found that students who overuse small electronic devices are more likely to experience wrist and hand pain, as well as changes to a particular nerve in their hands.^2^ In our study, the 61.94% who complained of neck and shoulder pain followed by tingling and numbness in the right hand obviously spent more than an hour on mobile devices and other electronic devices beyond their routine working hours. Therefore, we hope this novel study will raise awareness among the users of electronic devices of the importance of postural variation during their use. It also highlights the need for rest periods, so as to avoid prolonged use of such devices.

### Drawbacks

The major flaw and drawback in our study was we did not consider such complications as errors in diagnosis, non-operative management, and operative treatment options for the subjects with CTS symptoms in combination with neck pain and shoulder pain. An emphasis on prevention, resolution and treatment options should have been thoroughly implemented. In addition, threshold tests such as vibrometry and Semmes-Weinstein monofilaments, and innervation-density tests as suggested by Szabo et al^27^, should have been considered as non-invasive screening tests for CTS. Injection of the carpal tunnel with corticosteroid agents and wrist splinting^24^ are the principle components of non-operative treatment, which had to be considered and was not applied in our study. In our study we also did not consider EMG^6^, which is the typical gold standard measure for CTS confirmation.

## CONCLUSION

To conclude, a positive criterion for CTS with neck and shoulder pain was identified in our study with the usage of mobile devices for longer periods, rather than any ergonomic-related considerations in the working environment. It was recommended that 5 min of rest for every 30 min of continuous work and use of device was practised, and attention paid to the way that devices and instruments are held to help reduce symptoms. Further, combination tests like Phalen’s with pressure provocation tests proved accurate in conforming CTS. Individually in our study, the Tinel’s sign stood out as ineffective in ruling out CTS when compared with Phalen’s test. Future research is needed to confirm the diagnostic utility of these independent and combined clinical tests in less prevalent settings, including general practitioner clinics. Involvement of electronic devices come up in conversations with patients about hand pain. This needs further investigation in a larger, and better controlled studies to really establish a relationship, to definitively connect electronics use to carpal tunnel syndrome.

Future research should also address the effect of work-related psychosocial factors on symptoms of CTS. Also, a more complex classification should be used for both risk factor assessment and disease prevalence. Once these questions are answered, in order to ensure the success of adopted ergonomic jobs and workplace modifications, there should be an increase in workers’ awareness levels that will help the future job assessments. Only by combining jobs with ergonomic considerations, limiting the use of electronic devices, and using programmes that will reduce psychosocial stress levels will one obtain a real reduction in the number of claims in CTS.
